# Hepatocyte Isolation From Unused/Rejected Livers for Transplantation: Initial Step Toward Hepatocyte Transplantation, the First Experience From Iran

**DOI:** 10.5812/hepatmon.10397

**Published:** 2013-08-03

**Authors:** Mahdokht Hossein Aghdaie, Bita Geramizadeh, Negar Azarpira, Elahe Esfandiari, Masoume Darai, Marjan Rahsaz, Saman Nikeghbalian, Seyed Ali Malekhosseini

**Affiliations:** 1 Transplant Research Center, Shiraz University of Medical Sciences, Shiraz, IR Iran; 2 Department of Pathology, Shiraz University of Medical Sciences, Shiraz, IR Iran; 3 Transplant Ward, Shiraz University of Medical Sciences, Shiraz, IR Iran

**Keywords:** Hepatocyte, Liver, Transplantation

## Abstract

**Background:**

Hepatocyte transplantation is being used in patients with liver-based metabolic disorders and acute liver failure. Hepatocytes can be isolated from unused/rejected livers under sterile conditions.

**Objectives:**

The quality of the hepatocytes is very important and the main and initial step in hepatocyte transplantation is hepatocyte isolation. In this study we tried to set up the methods of hepatocyte isolation in order to use the high quality cells in acute liver failure or congenital metabolic disorders.

**Materials and Methods:**

In this study, during a year, hepatocytes were isolated from 7 unused/rejected livers among more than 300 harvested livers in Shiraz University of Medical Sciences. The two step collagenase perfusion method was used under GMP (Good manufacturing practice) for hepatocyte isolation.

**Results:**

Highly quality hepatocytes with high viability and low contamination were isolated. The mean viability was 71.8% ± 21.7. In the first 4 cases microbial contamination by Staphylococci, Diphtheroid and Klebsiella was detected, however the last 3 cases were free of any micro organisms. After 5 weeks of cryopreservation in -140°C, the cell viability was still acceptable.

**Conclusions:**

Hepatocyte isolation can be performed as the main and initial step for cell transplantation from unused/rejected liver. It is the first experience in Iran.

## 1. Background

Liver is an important organ where synthesis of proteins and metabolism of endogenous and exogenous toxins are performed (1). Orthotropic liver transplantation (OLT) has become the treatment of choice for end-stage liver diseases (2). This technique needs removal of the entire liver, so it needs a liver donor and shortage of liver donors and their timely availability has been always a world-wide problem. Death still occurs in patients with cirrhosis, acute liver failure or metabolic liver diseases whilst awaiting for liver transplantation (3). Therefore, techniques have been developed for human hepatocyte isolation and transplantation which is less invasive and can be immediately available (1). It is a bridge to OLT (Orthotropic Liver Transplantation) which has mostly been used in children with liver based inborn errors of metabolism for replacing a single deficient enzyme or its product, and in patients with acute liver failure in emergency situations, where the aim is to maintain liver function as a bridge to find a suitable liver for transplantation. The host liver is the ideal home for the hepatocytes, so all experiments have been performed by infusing cells through portal or splenic veins (1). The isolated hepatocytes can also be used for research projects such as drug developments and new therapeutic strategies (4). The first report of hepatocyte isolation has been by Berry and Friend in 1969 from rat liver (5). Then experiments for improving the hepatocyte isolation procedure from human liver were continued to increase isolated cells quality for transplantation (6). It took 10 years until the first attempt for hepatocyte transplantation was performed to treat experimentally induced acute liver failure and congenital enzymatic deficiencies in laboratory animals (7). However after these years, the reports of hepatocyte transplantations in human are still few and until now, according to the English literature, about 70 patients have been successfully transplanted by isolated human hepatocytes (about 40 cases with acute liver failure and 30 children with congenital enzyme deficiency) (4). Human hepatocytes for transplantation can be isolated from different sources such as liver tissues which have been rejected from liver transplantation (deceased donors), non-deceased resected livers and cirrhotic tissue (8). Innovative methods to improve cell quality and cryopreservation have been a hot topic of research. Shiraz Transplant Center is the leading center of liver transplantation in Iran, according to this opportunity we decided to start cell isolation from unused/rejected livers for OLT (Orthotropic Liver Transplantation), especially for using them in emergency situation of patients with acute liver failure to buy the patient time until a suitable donor liver is available for transplantation. This procedure needs sophisticated equipment and experienced staff, which has been prepared and trained in our center.

## 2. Objectives

In this report we will try to share our experience with hepatocyte isolation, from unused/rejected livers of deceased donors. This is the first report from Iran and the initial and most important step for obtaining high quality hepatocytes for future transplantation.

## 3. Materials and Methods

In this study human hepatocytes were isolated from harvested livers (after informed consent for donated livers) rejected/not used for transplantation due to different causes mostly because of severe steatosis. This means that these livers had poor quality for whole organ transplantation, but still yielded reasonable numbers of hepatocytes with good quality after isolation procedure for cell transplantation. During a year (September 2011 to October 2012), more than 300 livers from deceased donors have been harvested by transplant surgery team to be transplanted, among these 300 cases, 15 livers have been rejected and considered not suitable for transplantation. Between the 15 cases mentioned, seven livers were suitable for cell isolation, i.e. viral markers were negative, there were no laceration and etc. (Table 1).

**Table 1.** Characteristics of Rejected Livers Used for Hepatocyte Isolation

	Sex/Age	Cause of Death	Percentage of Steatosis	Cause of Rejection	Viability	Cell Numbers/gr Tissue	Recovered Microorganisms
**1**	female/54	intracranial hemorrhage	< 5%	portal vein thrombosis	50%	66 × 10^4^	staphylococci coagulase negative
**2**	female/43	cerebrovascular accident	> 60%	severe steatosis	44%	48 × 10^6^	staphylococci coagulase negative
**3**	female/56	intracranial hemorrhage	> 60%	severe steatosis	80%	2.3× 10^6^	diphteroids
**4**	male/47	intracranial hemorrhage	60%	severe steatosis	90%	7.5 × 10^6^	klebsiella aerogenes
**5**	female/50	cerebrovascular accident	60%	severe steatosis	73%	38.8 × 10^6^	-
**6**	female/35	cerebrovascular accident	60%	severe steatosis	65%	605 × 10^6^	-
**7**	female/50	subarachnoid hemorrhage	20%	portal vein thrombosis	60%	13.5 × 10^6^	-

**Figure 1.**
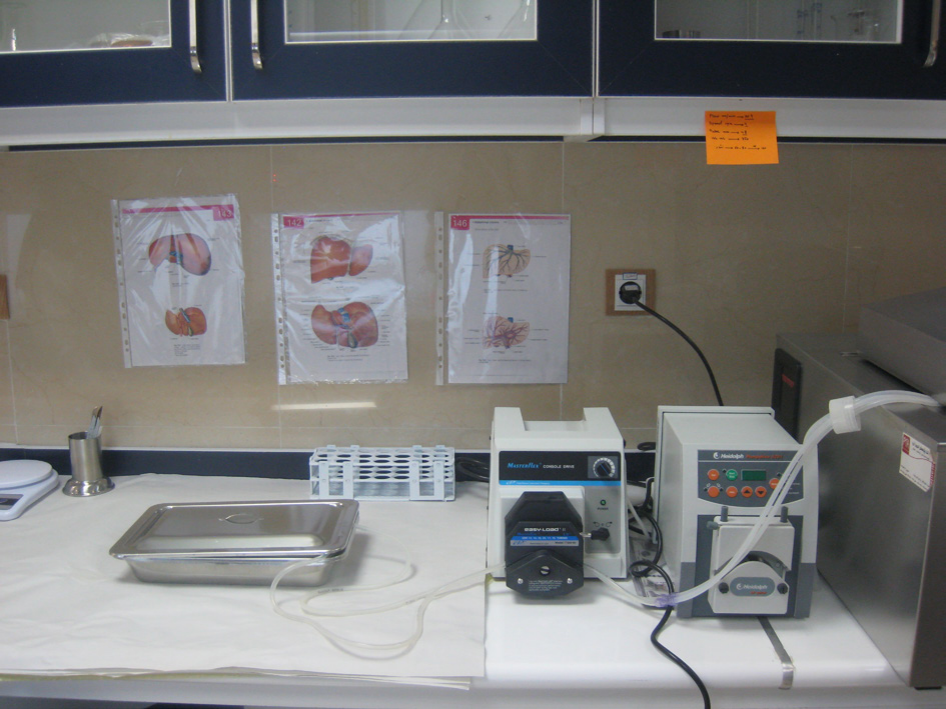
Main Bench of Hepatocyte Isolation Lab Shows Perfusion Pump

All the livers were processed within a few hours after harvesting. They were transferred to the hepatocyte isolation lab (Figure 1) on ice, in UW (University of Wisconsin) solution under sterile conditions. Human hepatocyte isolation was performed using collagenase perfusion with a two step technique with some modifications comparing to previous reports especially by using N-acetyl cysteine in steatotic livers, which has rarely been used in previous reports (5).

**Figure 2.**
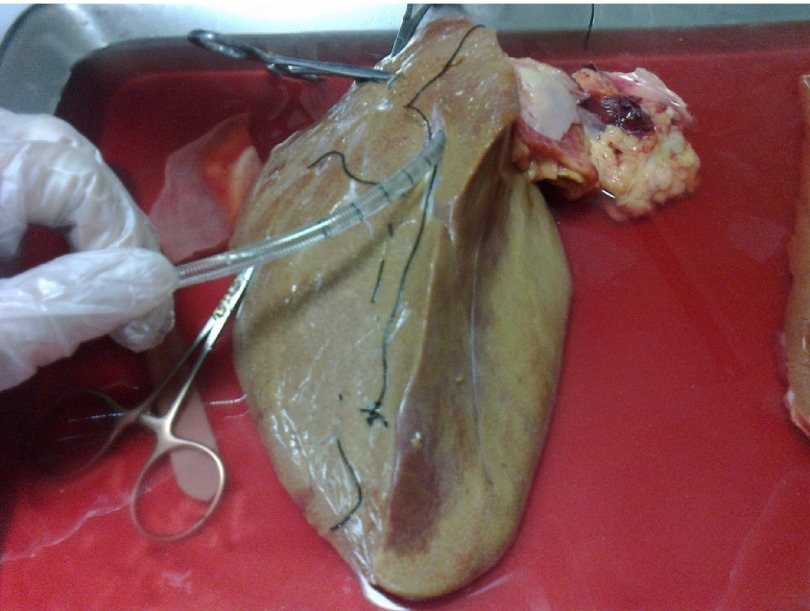
A Conulated Segment of Liver

**Figure 3.**
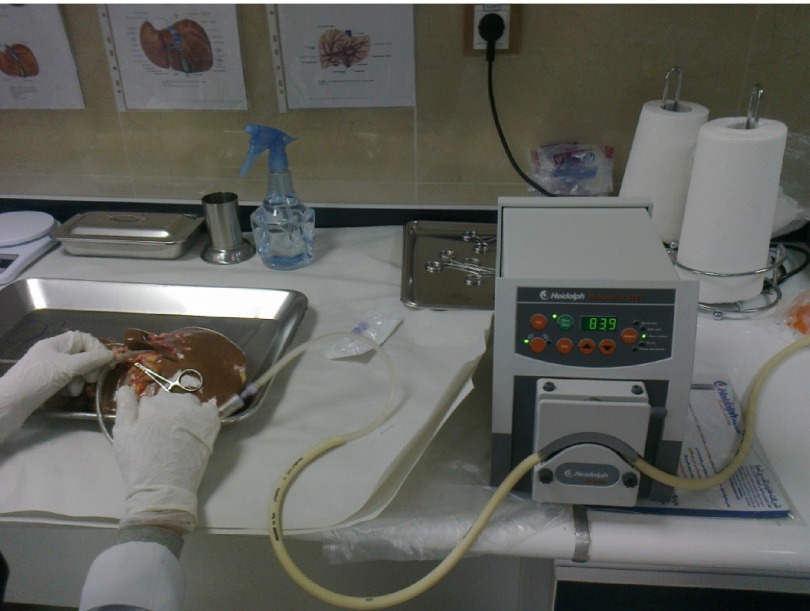
A Liver Cannulae Connected to Perfusion Pump

The liver was first washed by phosphate buffer solution (PBS, PH = 7.2) through the exposed cut vessels to remove remaining blood. We used segments of liver for perfusion by cannula through major vessels (Figure 2). Major vessels were conulated and the cannula secured by suturing. Small vessels not used for perfusion were sutured to prevent leakage during perfusion. The cannula were attached to the perfusion pump (Cole-Pulmer instrument company, UK), and set for 60 mL/min (Figure 3).

The liver was flushed through portal vein by perfusion solution at 37°C as below:

1) 500 cc calcium and magnesium free hanks balanced salt solution (HBSS) buffer, 0.5 mM EGTA (Ethylene Glycol Tetra acetic acid) and 5 mM N-acetyl cysteine.

2) Calcium and magnesium free Hank’s solution

3) Culture media (DMEM) and collagenase P (0.5 gr/Lit)

When digestion of the parenchyma could be visually detected, it was cut manually in small fragments in cold DMEM. The digested tissue (cell suspension) was centrifuged three times, and then the final sediment of hepatocytes was counted for the cell viability of the hepatocytes by hemocytometer and trypan blue exclusion technique (Figure 3). Although the whole process of isolation was performed in cell isolation lab under sterile condition GMP (Good Manufacturing Practice), microbial culture was also performed from the whole process from the harvested liver to cell isolates to detect any possibility for contamination especially during transportation. These cells can be used immediately for transplantation or cryopreserved in -140°C for future use. We have also performed this step for 5 weeks.

## 4. Results

During the study period from September 2011 to September 2012, there were 7 livers rejected from transplantation but still suitable for cell isolation, which were used for hepatocyte isolation in this study. All the isolated livers were from cadaveric livers, with mean age of 48.2 (ranging from 35 to 56 years). The cadavers were 6 females and one male (Table 1).

**Figure 4.**
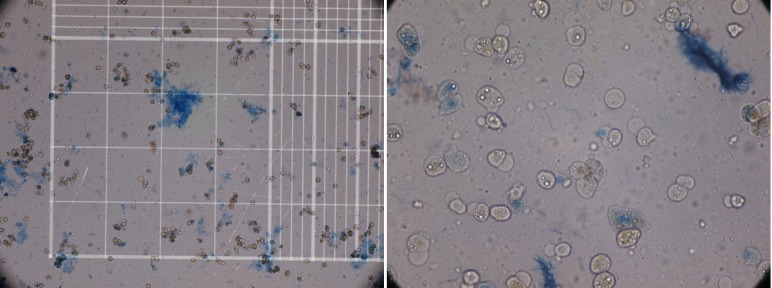
(left) Low and (right) High Power View of Trypan Blue Stained Cells Show Viable Cells with No Color. Dead Cells are Blue because they are Permeated by the Trypan Blue

As Table 1 shows the reason for rejected livers were macrovesicular steatosis more than 60% (Severe steatosis) and portal vein thrombosis. Causes of death in the deceased donors were shown in Table 1. The isolated cells showed mean viability of 71.8% ± 21.7 immediately after isolation by Try pan blue (Figure 4). The mean number of isolated cells was 155.3 × 10 ^6 ^± 50 × 10 ^6 ^cells. There was four isolates with microbial contamination, by coagulase negative Staphylococci, which is a skin commensal, Diphteroids and Klebsiella aero genes. All the isolated organisms were from the first step before isolation. After cryopreservation for 5 weeks, the cell viability was still high i.e. 75.1 ± 5% with no microbial isolate.

## 5. Discussion

Hepatocyte transplantation is a promising treatment as an alternative to liver transplantation (2). With cell transplantation, major surgery might be avoided and would be a possibility of using hepatocytes from livers that have been rejected from transplantation (9). Advantages of hepatocyte transplantation include maintaining the native liver in place which allows the possibility of native liver regeneration and the native liver serves as a back-up for transplanted cells, also the procedure is less invasive and the cryopreserved cells would be immediately available after cryopreservation in emergency situation (10). Human hepatocytes for cell transplantation have been isolated from different sources such as rejected/unused livers for transplantation (4), explanted cirrhotic livers (8) and normal tissue of resected livers in the patients with different hepatic metastasectomies such as metastatic colon cancer (8). Isolation of hepatocytes from different sources needs good experience to yield high quality cells with high viability to serve enough liver function for cell transplanted patient (4). The first hepatocyte isolation has been in mid-1960s, with two step collagenase perfusion technique, which is now widely used (3). In this technique the liver will be perfused by collagenase through a specific perfusion pump by cannula ting of the vessels. Then the cells will go through different steps of homogenization and cryopreservation (3). There has been significant progress in cell isolation and viability by several modifications to improve the quality of isolated cells, for example addition of N-acetylcysteine during isolation can improve cell viability especially in steatotic livers (10). Over a period of a year, in Shiraz liver transplant center, there have been more than 300 harvested livers and among these cases, we had the opportunity to isolate hepatocytes from 7 cadaveric livers that have been rejected from transplantation. Majority of these livers have been rejected because of high fat content. It is worthy to mention that we had 8 other rejected livers which have not been used for cell isolation because of different reasons such as technical problems and positive viral markers. As the Table 1 shows, seven mentioned livers were rejected because of high steatosis and portal vein thrombosis. After hepatocyte isolation there are very important steps to make sure that the isolated cells are suitable for transplantation (4). One of the most important criteria is cell viability (8). The precise amount of hepatocytes to maintain minimal metabolic requirement has not been definitely determined (11). There should be cell viability more than 60% and each infusion should be about 10 ^8^ cells (4). The mean cell viability in the isolated hepatocytes in our experience was 71.8% ± 21.7. The reports from UK and France have shown the mean viability of 60 ± 3.6% and 83.4 ± 1.0% respectively (12). Another major concern is obtaining cells free of any microbial contamination (13). Although there is no standard procedure for bacteriological study in hepatocyte transplantation, bacterial screening should be performed on the organ as it arrives and during the cell isolation procedure. The previous reports of the hepatocyte isolations have shown about 30% of contamination by organisms such as staphylococci, Diphtheroid and gram negatives as in our experience (13). All of our techniques of cell isolation have been performed under sterile condition, however, microbial cultures were positive in the first four isolated cases in the initial steps of cell isolation before perfusion of the organ, after considering the sterile transfer of the liver to the isolation lab, after harvesting, the last 3 cases were free of contamination. According to our experience, rejected/unused livers can be a good source of viable hepatocyte which can be used for cell transplantation in different situation. It is very important to isolate hepatocytes under GMP (Good manufacturing practice) conditions and also to isolate cells as soon as possible after harvesting of the liver. Both of these conditions need sophisticated equipment and experienced enthusiastic staff. After the hepatocyte isolation, the next step is cryopreservation of the isolated cells, because there is severe limitation in the number of donor livers that can be used for hepatocyte isolation (1).

Isolated hepatocytes have been used in two conditions:

1) Metabolic Diseases

Until now about 30 children and adults with various metabolic diseases such as Crigler-Najjar disease have received hepatocytes with acceptable short term recovery (14).

2) Acute Liver Failure

Hepatocyte transplantation can act as a bridge to help the patient survive to receive whole organ transplantation, because the cryopreserved hepatocytes are immediately available but liver for transplantation may not be available at once (14).

According to the previous studies, the standardization and optimization of hepatocyte isolation from fresh human liver is the most important step toward hepatocyte transplantation and should be established as a routine procedure (12). Our experience is the first step toward hepatocyte transplantation for the first time in Iran. The next plan is to use these isolated hepatocytes for transfusion in the patients with metabolic liver diseases or hepatic failure which will be reported in the future papers.
